# Metabolic Tumour Burden Measured by ^18^F-FDG PET/CT Predicts Malignant Transformation in Patients with Neurofibromatosis Type-1

**DOI:** 10.1371/journal.pone.0151809

**Published:** 2016-03-17

**Authors:** Axel Van Der Gucht, Ouidad Zehou, Soraya Djelbani-Ahmed, Laurence Valeyrie-Allanore, Nicolas Ortonne, Pierre Brugières, Pierre Wolkenstein, Alain Luciani, Alain Rahmouni, Emilie Sbidian, Emmanuel Itti

**Affiliations:** 1 Department of Nuclear Medicine, H. Mondor Hospital, Assistance Publique-Hôpitaux de Paris/Paris-Est University, Créteil, F-94010; 2 Department of Dermatology, H. Mondor Hospital, Assistance Publique-Hôpitaux de Paris/Paris-Est University, Créteil, F-94010; 3 Department of Pathology, H. Mondor Hospital, Assistance Publique-Hôpitaux de Paris/Paris-Est University, Créteil, F-94010; 4 Department of Neuroradiology, H. Mondor Hospital, Assistance Publique-Hôpitaux de Paris/Paris-Est University, Créteil, F-94010; 5 Department of Medical Imaging, H. Mondor Hospital, Assistance Publique-Hôpitaux de Paris/Paris-Est University, Créteil, F-94010; Institute of Biomedicine, FINLAND

## Abstract

**Background:**

To investigate the diagnostic and prognostic performances of ^18^F-FDG PET/CT measures of metabolic tumour burden in patients with neurofibromatosis type-1 (NF1), suspect of malignant transformation.

**Methods:**

This retrospective study included 49 patients (15–60 years old, 30 women) with a diagnosis of NF1, followed in our Reference Centre for Rare Neuromuscular Diseases, who presented clinical signs of tumour progression (pain, neurological deficit, tumour growth). Quantitative metabolic parameters were measured on 149 tumoral targets, using semi-automatic software and the best cut off values to predict transformation was assessed by Receiver Operating Characteristics (ROC) analysis. Prognostic value of PET/CT metabolic parameters was assessed by Kaplan-Meier estimates of overall survival.

**Results:**

Lesions were histologically documented in 40 patients: a sarcomatous transformation was found in 16, a dysplastic neurofibroma (NF) in 7, and a benign NF in 17; in the remaining 9 patients, a minimal follow-up of 12 mo (median 59 mo) confirmed the absence of transformation. The optimal cut off values for detection of malignant transformation were, in decreasing order of area under the ROC curves, a tumour-to-liver (T/L) ratio >2.5, SUV_max_ > 4.5, total lesion glycolysis (TLG) > 377, total metabolic tumour volume (TMTV) > 88 cm^3^, and heterogeneity index (HI_suv_) > 1.69. The best prognostic marker was the TLG: the 4-y estimates of survival were 97% [95% CI, 90% - 100%] in patients with TLG ≤ 377 vs. 27% [95% CI, 5% - 49%] in patients with TLG > 377 (P < 0.0001; χ^2^ 27.85; hazard ratio 13.27 [95% CI, 3.72–47.35]). T/L ratio, SUV_max_ and TMTV demonstrated slightly lower performance to predict survival, with χ^2^ ranging 14.41–19.12. The HI_suv_ index was not predictive of survival.

**Conclusion:**

Our study demonstrates that TLG and TMTV, as PET/CT measures of metabolic tumour burden, may be used clinically to identify sarcomatous transformation in patients with NF1 and predict overall survival, with a higher specificity for the TLG. Conventional measures such as the SUV_max_, and T/L ratio also demonstrate high prognostic value.

## Introduction

Neurofibromatosis type-1 (NF1) is one of the most common autosomal dominant genetic diseases involving the neurofibromin gene on chromosome 17q11.2 [1]. Although dermatological lesions are the leading clinical feature, NF1 is multi-system disease and is associated with a high risk of developing cancers (4 times higher than in general population) [2,3]. The outcome is largely dominated by brain cancer, vascular disease, and the development of malignant peripheral nerve sheath tumours (MPNSTs) [4,5]. MPNSTs usually arise from pre-existing internal neurofibromas (NFs) and are associated with a median overall survival of 24 months [2]. Differentiating between benign and malignant tumours has important prognostic and therapeutic implications, but can be difficult, especially in individuals with multiple benign tumors. The clinical manifestations of malignancy include unremitting pain not otherwise explained, rapid increase in size of a plexiform neurofibroma, change in consistency, and new or unexplained neurological deficit [6]. Furthermore, diagnosis of MPNSTs is challenging because some deep lesions are not accessible to clinical examination or biopsy and magnetic resonance imaging (MRI) provides site and extent information, has variable diagnostic performance, with 43–63% sensitivity and 67–100% specificity [7,8]. Therefore, an at-risk phenotype of morbid-mortality has been defined [9,10] consisting of a prediction score of internal tumour burden, linking the number of subcutaneous neurofibromas to internal neurofibromas at risk of dysplasia (characterized by the association of hypercellularity and cytonuclear atypia [11]) and malignant transformation.

Positron emission tomography with ^18^F-fluorodeoxyglucose (^18^F-FDG PET) is the most frequently used functional imaging procedure for evaluating tumour glucose metabolism and proliferation [12]. It is increasingly used for the management of patients with NF1, particularly for the detection of MPNSTs, as glucose metabolism in sarcomas is increased in proportion to their higher proliferative index, undifferentiating grade and metastatic spread [12–14]. Compared to whole-body MRI, Derlin et al. [15] showed a sensitivity of close to 100% in the detection of MPNSTs (against 67% with MRI). It has been reported that a maximum standardized uptake value (SUV_max_) > 2.5 on early imaging and > 3.5 on delayed imaging can accurately predict malignant transformation with positive and negative predictive values of 50–95% and 82–100%, respectively [13,16]. In recent series, other semi-quantitative indices have been proposed, such as the intratumoral heterogeneity index (HI_suv_) [17], the tumour-to-liver (T/L) uptake ratio [18,19], and the SUV derived for lean body mass [20]. Metabolic tumour volume (MTV) and total lesion glycolysis (TLG) have been proposed to overcome the limited representative nature of SUV_max_ and to reflect the volume of the tumor [21,22]. Many studies in head-and-neck cancer, non-small-cell lung cancer and epithelial ovarian cancer have consistently demonstrated the superior prognostic value of MTV and TLG compared with SUV_max_ [23–25]. In particular, it has been shown a reliable prognostic factor in soft-tissue sarcomas [26], and a valuable management tool in patients with MPNSTs [27].

Aim of the current study was to investigate from a single population the diagnostic and prognostic performances of several known measures of metabolic tumour burden (SUV_max_, HI_suv_, T/L ratio, MTV and TLG), assessed by whole-body ^18^F-FDG PET/CT, in NF1 patients suspected to undergo malignant transformation.

## Materials and Methods

### Study population

This retrospective study included all consecutive patients followed in our Reference Centre for Rare Diseases, with a diagnosis of NF1 according to the National Institute of Health (NIH) criteria [28], and with clinical manifestations that were suspected to undergo malignant transformation into MPNST (pain, neurological deficit, tumour growth). All patients underwent imaging in accordance with current national regulations (verbal informed consent was obtained and reported in patient medical records). Collection and analysis of data were retrospective and performed after de-identification. This non-interventional study was approved by our IRB on March 3, 2014 (Comité de Protection des Personnes Ile-de-France VI), and written consent was waived.

### PET/CT imaging

All referred patients underwent whole-body PET/CT imaging on a Gemini GXL 16 instrument (Philips, Da Best, The Netherlands) 50–60 minutes after intravenous injection of 5 MBq/kg ^18^F-FDG. They were required to fast for at least 6 h before undergoing the scan. The blood glucose level was previously verified with a dedicated electronic device so as to ensure a level of <1.5 g.l^-1^. A low-dose helical CT (120kV, 80–100 mAs) was first performed for anatomical correlation and attenuation correction. Then, whole-body emission images were acquired using 11–15 overlapping bed positions of 2 min each, and reconstructed using a line of response-row action maximum likelihood algorithm (2 iterations, 28 subsets, postfilter 5.1mm), with and without CT attenuation correction (matrix size of 128 x 128, voxel size 4 x 4 x 4 mm^3^). Evaluation of images was performed by two nuclear medicine physicians, blinded to histopathological results.

### Quantitative metabolic parameters

**Fig 1:**
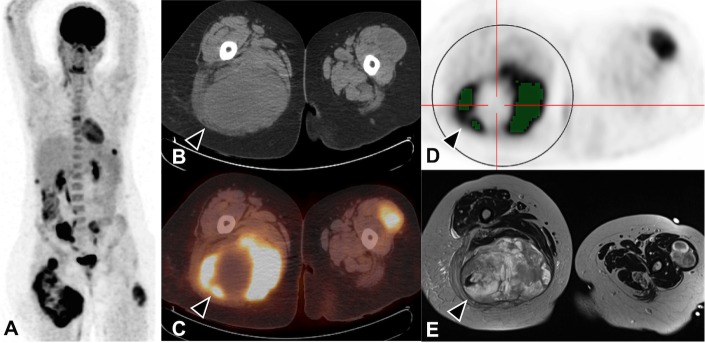
Example of a 19-year-old woman with rapidly growing peripheral neurofibroma in the upper right thigh ("at-risk" score = 25). (A) Maximum intensity projection. (B) CT image in axial view. (C) PET/CT fusion. (D) ^18^F-FDG PET images after drawing a spherical volume-of-interest around the intramuscular tumour: the metabolic volume corresponds to the sum of voxels above a 41% SUV_max_ threshold (green color). (E) Contrast-enhanced MRI. Metabolic measures suggest MPNST transformation (SUV_max_ = 12.5, TMTV = 389 cm^3^, TLG = 2323, T/L ratio = 6.2), confirmed by histopathological study (sarcoma grade 3). To note, intratumoral heterogeneity is present in the right thigh mass (black arrow) although whole-body heterogeneity index is below the predictive threshold (HI_suv_ = 1.68). Patient was treated with m-TOR inhibitors and died 6 months later.

Our approach was to characterize global metabolic tumour burden by different PET-derived parameters. First, SUV_max_ of each hypermetabolic NF1 tumour was identified visually on the attenuation-corrected. Then, a spherical, cylindrical or cubical bounding volume-of-interest was drawn around each target lesion by a nuclear medicine physician (SDA); shape and size of the volumes-of-interest were adjusted to ensure that they neither included areas of high physiological uptake (bladder, myocardium) nor exceeded the anatomical tumour boundaries seen on CT in case of low tumour/background contrast. Metabolic tumour volume (MTV) was computed within each volume-of-interest by summing all voxels above a 41% SUV_max_ threshold using a semiautomatic software (Imagys®; Keosys, Saint-Herblain, France) [29]. SUV_mean_ was also computed within each thresholded volume-of-interest. Total MTV (TMTV, expressed in cm^3^) was calculated by summing the MTV of all target lesions [30]. Total lesion glycolysis (TLG) was computed by summing the MTV × SUV_mean_ products of all volumes-of-interest. The heterogeneity index (HI_suv_) was calculated by dividing intratumoral SUV_max_ by SUV_mean_, and the tumour-to-liver (T/L) ratio by dividing intratumoral SUV_max_ by SUV_mean_ of the healthy liver [19]. An example is illustrated in Fig 1. Arbitrarily, in patients where no hypermetabolic target could be identified, quantitative parameters were set to 0.

### Statistical analyses

Receiver-operating characteristics (ROC) analysis was performed to determine optimal cut off values of PET/CT metabolic measures for the detection of malignant transformation. Continuous variables were expressed as median with their respective range. Categorical variables were presented with absolute and relative frequencies. Comparison of metabolic variables within the different clinical and histopathological categories was performed using the Mann-Whitney rank sum test. The prognostic value of PET/CT metabolic measures was assessed using overall survival (calculated from the date of diagnosis until death from cancer) as the end-point, with censoring at the time of last follow-up. Survival curves were obtained from Kaplan-Meier estimates and compared using the log-rank test. A *P*-value < 0.01 was considered significant (Bonferroni correction, 5 metabolic variables).

## Results

### Patient characteristics

Between 2006 and 2012, 49 NF1 patients (30 women, mean age 32.8 ± 12 y) with 149 tumoral targets were included (median 2 lesions/patient, extremes 0–9). Clinical and demographic data are summarized in Table 1. Forty lesions in 40 patients were histologically documented, of which 16 demonstrated a sarcomatous transformation, 7 a dysplastic NF, and 17 a benign NF; in the remaining 9 patients, a minimal follow-up of 12 mo (median 59 mo) confirmed the absence of sarcomatous transformation. Also, PET/CT revealed incidental tumor in 3 patients, a gastro-intestinal stromal tumor in one patient and a thyroid nodule in 2 patients (which were benign at fine needle aspiration). These foci of uptake were not considered as targets and were excluded from metabolic quantitative measures. The median delay between clinical symptoms and PET/CT imaging was 6.3 mo (extremes 0–42 mo). The concordance between the symptomatic lesion and the most hypermetabolic lesion on PET was found in 65% of patients. Median follow-up of living patients was 50 mo (extremes 0–102 mo) and 14 patients died from their disease at a median of 8.6 months (3–86 mo). Patients with MPNST were treated by surgery (n = 8), chemotherapy (n = 4), targeted therapy (n = 15) or radiotherapy (n = 7), with an association of 2 or 3 treatment modalities in 11 of them. All relevant data are provided in S1 Table.

**Table 1:** Clinical characteristics.

Characteristics	Value
Subjects, *n*	49
Gender, *n* (%)	
Male	19 (39)
Female	30 (61)
Age at diagnosis (mean, standard deviation)	32.8 ± 12
Age ≤ 30* *n* (%)	27 (55)
Family history of NFs, *n* (%)	27 (55)
Cutaneous manifestations, *n* (%)	
Absence of cutaneous NFs*	10 (20)
≥ 2 subcutaneous NFs*	35 (71)
Plexiform NFs*	32 (65)
< 6 café-au-lait spots	9 (18%)
Symptoms, *n* (%)	
Pain	45 (92)
Neurological deficit	18 (37)
Tumour growth	18 (37)
Histopathological evaluation, *n*	
MPNSTs	
High-grade	13
Intermediate-grade	2
Unknown-grade	1
Benign NF	17
Dysplastic NF	7

* clinical features included in the "at-risk" phenotype

### Univariate analysis of metabolic parameters within the different clinical and histopathological categories

**Fig 2:**
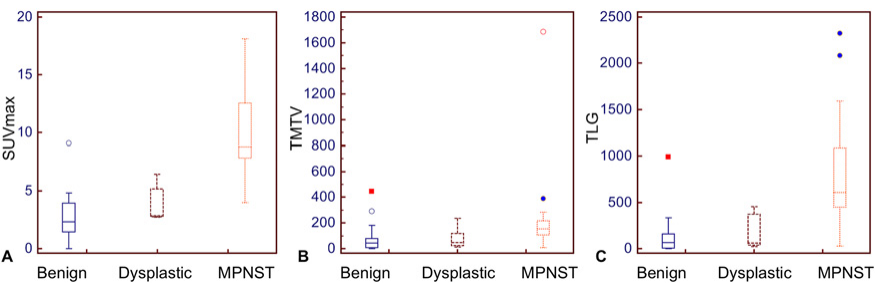
Box-plot repartition of SUV_max_ (A), TMTV (B) and TLG (C) in the three histopathological groups of tumours.

Box-plot repartition of SUV_max_, TMTV and TLG in the three histopathological groups of tumours is given in Fig 2. Results of the Mann-Whitney rank sum test are displayed in Table 2. All metabolic parameters but the HI_suv_ were significantly higher in the subgroup of patients with tumour growth (n = 18) compared to the subgroup without tumour growth, with *P*-values ranging 0.002 for TLG (median, 509 vs. 60, respectively) to 0.0002 for the T/L ratio (3.3 vs. 1.4, respectively). In addition, all metabolic parameters but the HI_suv_ were significantly higher in the subgroup of patients with histologically proven sarcoma (n = 16) compared to the remaining patients with benign or dysplastic NF, with P-values ranging 0.003 for TMTV (154 cm^3^ vs. 51 cm^3^, respectively) to < 0.0001 for SUV_max_ (8.8 vs. 2.9, respectively), TLG (609 vs. 69, respectively) and T/L ratio (4.9 vs. 1.5, respectively). No significant differences of metabolic parameters were found between patients with benign NF vs. patients with dysplastic NF. No significant differences were found between patients according to gender, age, family history, dermatological manifestations, pain and neurological symptoms; a trend for higher HI_suv_ was seen in patients with cutaneous NFs vs. patients without cutaneous NFs (1.71 vs. 1.65, *P* = 0.02).

**Table 2:** Univariate analyses of all population.

Variable		SUV_max_	*P*	TMTV (cm^3^)	*P*	TLG	*P*	HI_suv_	*P*	T/L ratio	*P*
		Median [range]		Median [range]		Median [range]		Median [range]		Median [range]	
Gender	Male	4.0 [0–15.6]	NS	96 [0–1687]	NS	232 [0–2083]	NS	1.73 [0–1.82]	NS	1.9 [0–6.3]	NS
	Female	3.8 [0–18.1]		55 [0–389]		119 [0–2323]		1.69 [0–1.85]		1.6 [0–10.1]	
Age	> 30	5.1 [0–15.6]	NS	106 [0–444]	NS	311 [0–1129]	NS	1.72 [0–1.81]	NS	2.2 [0–7.7]	NS
	≤ 30*	2.9 [0–18.1]		53 [0–1687]		85 [0–2323]		1.70 [0–1.85]		1.5 [0–10.1]	
Family history	No	5.3 [0–18.1]	NS	147 [0–389]	NS	311 [0–2323]	NS	1.72 [0–1.85]	NS	2.3 [0–10.1]	NS
	Yes*	2.9 [0–15.6]		53 [0–1687]		85 [0–2083]		1.71 [0–1.84]		1.5 [0–8.3]	
Cutaneous NF	Yes	4.0 [0–18.1]	NS	78 [0–1687]	NS	157 [0–2323]	NS	1.71 [0–1.85]	0.02	1.9 [0–10.1]	NS
	No*	2.3 [0–12.6]		83 [0–237]		208 [0–834]		1.65 [0–1.73]		1.2 [0–8.3]	
Subcutaneous NF	< 2	3.0 [0–11.2]	NS	50 [0–291]	NS	86 [0–1130]	NS	1.71 [0–1.85]	NS	1.5 [0–5.8]	NS
	≥ 2*	4.1 [0–18.1]		90 [0–1687]		228 [0–2323]		1.71 [0–1.84]		1.9 [0–10.1]	
Plexiform NF	No	2.8 [0–14.6]	NS	76 [0–291]	NS	170 [0–1044]	NS	1.70 [0–1.78]	NS	1.5 [0–8.3]	NS
	Yes*	4.0 [0–18.1]		79 [0–1687]		142 [0–2323]		1.71 [0–1.85]		1.8 [0–10.1]	
Café-au-lait spots	≥ 6	4.0 [0–18.1]	NS	82 [0–1687]	NS	158 [0–2323]	NS	1.71 [0–1.85]	NS	1.9 [0–10.1]	NS
	< 6*	2.9 [0–15.6]		43 [0–237]		70 [0–599]		1.69 [0–1.81]		1.5 [0–6.3]	
Pain	No	4.2 [0–4.8]	NS	48 [0–1687]	NS	106 [0–2083]	NS	1.71 [0–1.74]	NS	2.1 [0–4.4]	NS
	Yes	3.9 [0–18.1]		85 [0–444]		159 [0–2323]		1.71 [0–1.85]		1.6 [0–10.1]	
Neurological deficit	No	3.9 [0–18.1]	NS	57 [0–1687]	NS	111 [0–2083]	NS	1.71 [0–1.85]	NS	1.6 [0–10.1]	NS
	Yes	4.0 [0–14.6]		123 [0–444]		347 [0–2323]		1.70 [0–1.84]		1.9 [0–8.3]	
Tumour growth	No	2.8 [0–18.1]	0.0003	47 [0–444]	0.009	60 [0–1592]	0.002	1.67 [0–1.85]	0.05	1.4 [0–10.1]	0.0002
	Yes	7.8 [1.6–15.6]		148 [6–1687]		509 [8–2323]		1.73 [1.44–1.82]		3.3 [0.7–8.3]	
MPNST	No	2.9 [1.5–9.1]	<0.0001	51 [7–444]	0.003	69 [8–990]	<0.0001	1.69 [1.44–1.85]	NS	1.5 [0.7–3.9]	<0.0001
	Yes	8.8 [4.0–18.1]		154 [6–1687]		609 [28–2323]		1.73 [1.65–1.81]		4.9 [2.2–10.1]	
NF type	Benign	2.9 [1.5–9.1]	NS	53 [7–444]	NS	70 [8–990]	NS	1.69 [1.44–1.84]	NS	1.5 [0.7–3.9]	NS
	Dysplastic	2.9 [2.7–6.4]		47 [12–237]		58 [20–452]		1.68 [1.63–1.85]		1.5 [1.3–3.3]	

* clinical features included in the “at-risk” phenotype

### ROC curve analysis

As detailed in Table 3, ROC analysis revealed that the performance of metabolic parameters for the detection of malignant transformation was, in decreasing order of area under the ROC curve (AUC), the T/L ratio, the SUV_max_, the TLG, the TMTV and the HI_suv_. The optimal cut off values were T/L ratio >2.5, SUV_max_ > 4.5, TLG > 377, TMTV > 88 cm^3^, and HI_suv_ > 1.69, respectively. When analysis was restricted to patients with an histologically-proven diagnosis (n = 40), these cut off values were similar, T/L ratio >2.5, SUV_max_ > 4.4, TLG > 377, TMTV > 87 cm^3^, and HI_suv_ > 1.70, respectively, but with slightly lower AUC values.

**Table 3:** Performance of PET/CT metabolic parameters to distinguish sarcoma from benign NF, dysplastic NF and no sign of transformation at follow-up (n = 49).

Characteristics	SUV_max_	TMTV (cm^3^)	TLG	HI_suv_	T/L ratio
Median [range]	3.9	80	157	1.71	1.8
	[0–18.1]	[0–1687]	[0–2323]	[0–1.85]	[0–10.1]
Cut off	> 4.5	> 88	> 377	> 1.69	> 2.5
Sensibility (%)	94	94	81	81	94
Specificity (%)	88	79	94	58	94
AUC [95% CI]	0.96	0.82	0.91	0.67	0.98
	[0.87–1.00]	[0.69–0.92]	[0.80–0.97]	[0.52–0.80]	[0.89–1.00]
*P*-value	< 0.0001	< 0.0001	< 0.0001	0.03	< 0.0001

### Survival analysis

**Fig 3:**
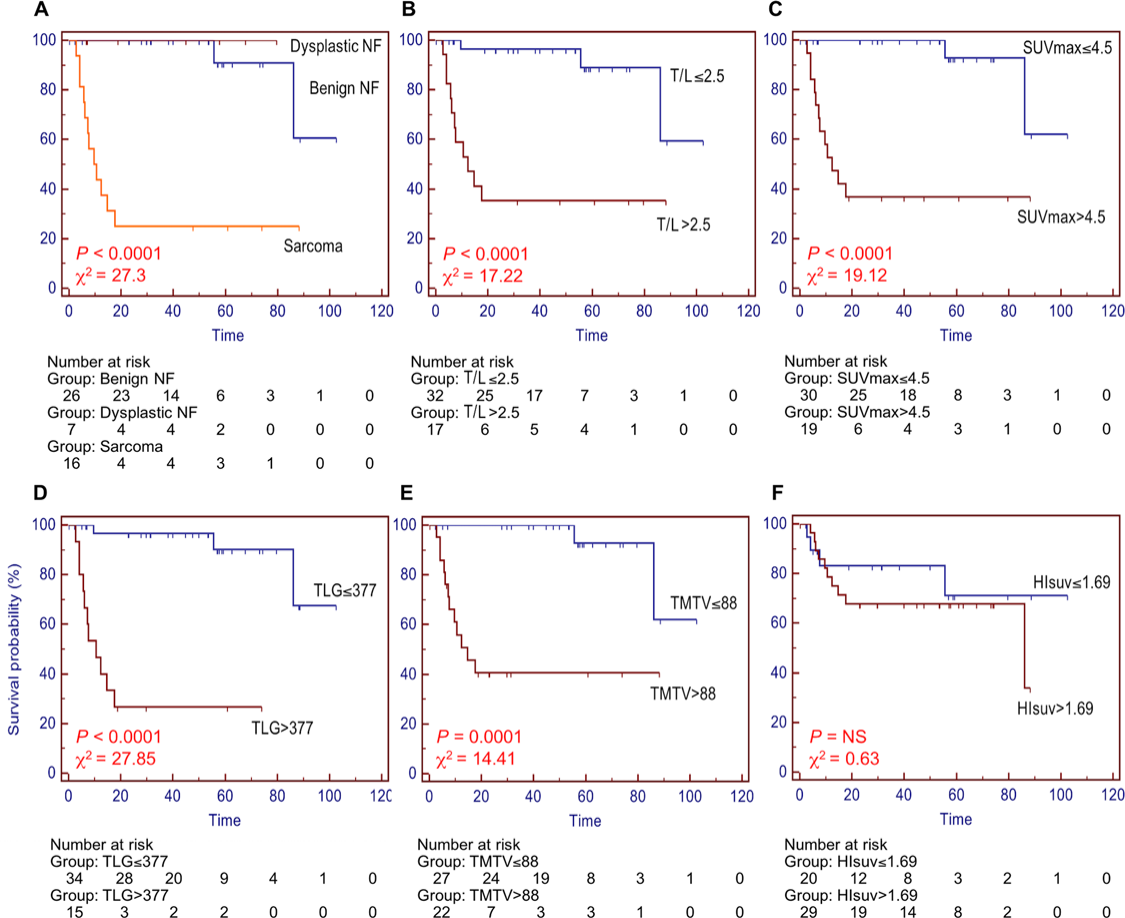
Kaplan-Meier estimates of overall survival with number of patients at risk at different time points depending on histopathology (A) and metabolic measures: T/L ratio (B), SUV_max_ (C), TLG (D), TMTV (E), HI_suv_ (F) calculated in the whole population (n = 49, median follow-up 50 mo).

With a median follow-up of 50 mo, the 4-y survival was 100% [95% CI, 100% - 100%] in patients with benign NF or no sign of transformation at follow-up, 100% [95% CI, 100% - 100%] in patients with a dysplastic NF and 25% [95% CI, 4% - 46%] in patients with sarcoma transformation (P < 0.0001, Fig 3A). The same survival estimates were obtained in the subset of patients with histologically-proven diagnosis (P < 0.001). The performances of PET/CT metabolic parameters to predict survival are given in Fig 3B–3F. The best prognostic marker was the TLG: the 4-y estimates of survival were 97% [95% CI, 90% - 100%] in patients with TLG ≤ 377 vs. 27% [95% CI, 5% - 49%] in patients with TLG > 377 (P < 0.0001; χ^2^ 27.85; hazard ratio 13.27 [95% CI, 3.72–47.35]). T/L ratio, SUV_max_ and TMTV demonstrated slightly lower performance to predict survival, with χ^2^ ranging 14.41–19.12. The HI_suv_ index was not predictive of survival.

## Discussion

Aim of the current study was to investigate from a single population the diagnostic and prognostic performances of several known measures of metabolic tumour burden (SUV_max_, HI_suv_, T/L ratio, MTV and TLG), assessed by whole-body ^18^F-FDG PET/CT, in NF1 patients suspected to undergo malignant transformation. This study confirmed the prognostic value of PET/CT measures of metabolic tumour burden in patients with NF1. All metabolic parameters, except the HI_suv_, were significantly higher in patients with histologically-proven sarcoma, compared with the remaining patients with benign or dysplastic neurofibromas. In particular, TLG, which reflects both the tumour burden and the proliferation rate, appeared the best prognosticator in our series, with a cut off > 377, while TMTV was slightly less predictive of survival with a cut off > 88 cm^3^.

Brenner et al. found that a SUV_max_ > 3.0 was a significant predictor of poor survival in 16 NF1 patients with MPNST [31]. Choi et al. [26] have recently shown that TLG was a more accurate predictor of progression-free survival than SUV_max_ or MTV in 66 patients with soft-tissue sarcoma of various histologies (including 6 patients with MPNST), with areas under the ROC curves of 0.802, 0.726 and 0.681, respectively. The cut off values for disease progression derived from these data were TLG > 250, SUV_max_ > 6.0, and MTV > 40 cm^3^, but cut offs for the specific subset of patients with MPNST was not reported. TLG was found to allow preoperative assessment of disease progression with an accuracy comparable to that obtained with conventional well-known clinicopathological variables, such as the American Joint Committee of Cancer (AJCC) stage, Fédération Nationale des Centres de Lutte Contre le Cancer (FNCLCC) grade, tumour size, and involvement of the surgical margin. This may further explain why TLG was the best prognostic biomarker in our series, as it combines morphological (tumour volume) and metabolic information (presumably correlated to FLNCCC items, namely the mitotic index, undifferentiation and intratumoral necrosis). Khiewvan et al. [27] have also shown in a retrospective study of 21 patients with MPNST who underwent PET/CT imaging both before and after treatment, that TLG and MTV were prognostic factors for survival, whereas SUV_max_ and SUV_mean_ were not. Our results differ from this study probably due to different patient stages, SUV ranges, and follow-up times. In particular, Khiewvan et al. [27] had a longer follow-up time (range 7–216 months) than ours (range 0–102) and than in the study by Brenner et al. (range 5–62 months). TLG has also demonstrated its incremental prognostic value in other types of cancer such as renal cell carcinoma, lung cancer, ovarian cancer, head-and-neck cancer and osteosarcomas [23–25,32].

The performance of SUV_max_ and T/L ratio to predict MPNST transformation in NF1 patients has been previously reported in several studies, although the cut off values vary a lot from one study to the other, ranging > 2.5 to > 7.0. In a recent report of 50 patients, a SUV_max_ cut off value > 4.1 (AUC = 0.98) [17] was used to separate malignant from benign tumours, with a sensitivity of 100% and a specificity of 87%, but no survival analysis was performed. Our cut off value of SUV_max_ > 4.5 (AUC = 0.96) is consistent with these findings, with comparable sensitivity and specificity, 94% and 88%, respectively. The same team has reported that the T/L ratio was even more predictive of MPNST transformation than SUV_max_, with a cut off value > 2.6 [19], with similar sensitivity but with a greater specificity (90% for T/L ratio vs. 87% for SUV_max_). In our study, with a ROC-derived cut off value > 2.5, we also achieved a greater specificity using T/L ratio, compared with SUV_max_ (94% vs. 88%, respectively), with same sensitivity (94% vs. 94%, respectively). As a fact, T/L ratio was the best metabolic parameter to predict sarcoma transformation with the highest AUC (0.98). Furthermore, the heterogeneity index was investigated by the same authors in a population of 19 MPNSTs diagnosed in 50 NF1 patients. Uptake heterogeneity was graded qualitatively using a 3-point scale (0 = homogeneous uptake, 1 = areas of focally increased uptake in < 33% of a lesion as compared to the background uptake within that lesion, 2 = areas of increased uptake in > 33% of a lesion) and semi-quantitatively by calculating the HI_suv_ index. A cut off value > 1.4 (AUC = 0.76) provided a sensitivity of 100% and a specificity of 23%. Quantitative HI_suv_ demonstrated a highly significant correlation with visual heterogeneity (*r* = 0.5, p < 0.0001). In our series, HI_suv_ showed a lower sensitivity of 81%, but a higher specificity of 58% for a HI_suv_ cut off > 1.69. The visual heterogeneity was not evaluated here.

Furthermore, the radiation dosage of ^18^F-FDG PET/CT can be discussed. CT has been an increasing source of radiation exposure during the past decades. Epidemiological data suggest that diagnostic imaging using ionizing radiations may increase the risk of cancer in developed countries by 0.6–3% [33]. However, these data rely on the assumption that there is a linear no-threshold relationship between radiation dose and carcinogenic effects, which is uncertain under 1 Gy [34]. In contrast, experimental data suggest that cell defenses are more efficient at a low dose and low dose rate, and that DNA repair or cell death (at a very low dose rate) may eliminate the damage [34]. The effective dose of a PET/CT in our study was around 15 mSv, that is less than a full-dose diagnostic CT of thorax-abdomen-pelvis.

The present study has several limitations. It is a retrospective study with a relatively limited number of patients. However, to our knowledge this is the first study to date comparing the performance of TMTV and TLG in NF1 patients with clinical manifestations that were suspected to undergo malignant transformation.

Our results are monocentric as all PET/CT exams were performed using the same camera, in the same centre, and with the same standardized acquisition protocol. Furthermore, the segmentation method for metabolic volume computation might be discussed. Compared to studies of Choi et al. [26] and Khiewvan et al. [27], which have used 40% and 45% SUV_max_ thresholds, respectively, the segmentation method used in the current study was a fixed 41% SUV_max_ threshold. It has been shown and confirmed by recent phantom study [29] and clinical studies [35,36] that this method gives the best prognostic estimate of the tumoral burden in Hodgkin and non-Hodgkin lymphomas. The fixed 41% SUV_max_ threshold method has been recommended by the latest European Association of Nuclear Medicine guidelines [30]. Finally, the HI_suv_ was not predictive of malignant transformation in our series, probably because we used an averaged value between up to 9 lesions per patient (in order to have a global characterization of tumour metabolic burden).

## Conclusion

In conclusion, the current study demonstrates that TLG and TMTV, which are measures of metabolic tumour burden assessed by PET/CT, may be used clinically to identify MPNST transformation and predict overall survival, with a higher specificity for the TLG. Conventional measures such as the SUV_max_, and T/L ratio also demonstrate high prognostic value. These PET/CT quantitative measures were consistent with clinical manifestations and histopathological features in NF1. Further studies are warranted to confirm our results in a prospective design.

## Supporting Information

S1 Table All relevant data. (DOCX)
