# Supplementary material for: Metabolic Tumour Burden Measured by 18F-FDG PET/CT Predicts Malignant Transformation in Patients with Neurofibromatosis Type-1
Source: PLoS One. 2016 Mar 17;11(3):e0151809. doi: 10.1371/journal.pone.0151809 (PMC4795780; doi:10.1371/journal.pone.0151809)
Supplement: S1 Table — (DOCX) [file pone.0151809.s001.docx]

| Patient | Gender (0=man) | Age | MTV Total | TLG Total | SUVmax | Liver | T/L ratio | HIsuv | Death (1=yes) | Survival |
| --- | --- | --- | --- | --- | --- | --- | --- | --- | --- | --- |
| 1 | 1 | 47 | 181,0 | 294,4 | 4,1 | 2,1 | 22,4 | 1,8 | 0 | 23,0 |
| 2 | 1 | 21 | 236,7 | 376,6 | 2,7 | 2,1 | 10,0 | 1,7 | 0 | 6,6 |
| 3 | 1 | 55 | 46,8 | 58,4 | 2,8 | 2,0 | 27,5 | 1,6 | 0 | 7,0 |
| 4 | 0 | 44 | 291,0 | 331,7 | 2,0 | 1,8 | 24,4 | 1,7 | 0 | 23,2 |
| 5 | 0 | 32 | 148,2 | 717,4 | 8,0 | 1,6 | 19,6 | 1,7 | 1 | 2,8 |
| 6 | 0 | 37 | 15,7 | 40,1 | 4,3 | 2,3 | 16,4 | 1,7 | 0 | 2,3 |
| 7 | 0 | 53 | 283,8 | 1129,7 | 11,2 | 1,9 | 27,7 | 1,8 | 1 | 17,7 |
| 8 | 0 | 37 | 78,3 | 122,3 | 3,3 | 2,2 | 16,8 | 1,7 | 0 | 38,2 |
| 9 | 1 | 27 | 159,3 | 617,5 | 10,0 | 1,6 | 16,6 | 1,7 | 1 | 4,0 |
| 10 | 1 | 21 | 43,0 | 70,1 | 2,9 | 2,0 | 10,5 | 1,8 | 0 | 53,4 |
| 11 | 1 | 60 | 89,8 | 290,4 | 8,4 | 2,8 | 21,5 | 1,7 | 0 | 88,3 |
| 12 | 1 | 47 | 128,5 | 452,1 | 5,9 | 2,4 | 19,6 | 1,7 | 0 | 18,8 |
| 13 | 1 | 19 | 389,2 | 2323,2 | 12,5 | 2,0 | 9,4 | 1,7 | 1 | 4,2 |
| 14 | 1 | 39 | 6,0 | 27,9 | 7,9 | 2,4 | 16,3 | 1,7 | 0 | 47,6 |
| 15 | 0 | 19 | 95,6 | 228,3 | 4,8 | 2,2 | 8,6 | 1,8 | 1 | 9,6 |
| 16 | 1 | 31 | 118,3 | 399,0 | 8,3 | 2,0 | 15,5 | 1,7 | 1 | 10,6 |
| 17 | 1 | 49 | 13,8 | 14,9 | 1,7 | 2,1 | 23,9 | 1,6 | 0 | 58,7 |
| 18 | 1 | 31 | 8,9 | 8,1 | 1,5 | 2,0 | 15,3 | 1,6 | 0 | 57,0 |
| 19 | 1 | 25 | 75,5 | 157,0 | 4,4 | 2,7 | 9,2 | 1,7 | 0 | 27,8 |
| 20 | 1 | 31 | 12,5 | 12,0 | 1,9 | 1,9 | 16,8 | 1,8 | 0 | 29,8 |
| 21 | 1 | 25 | 87,5 | 347,9 | 6,4 | 2,0 | 12,9 | 1,6 | 0 | 79,6 |
| 22 | 1 | 28 | 0,0 | 0,0 | 0,0 | 2,0 | 14,1 | 0,0 | 0 | 88,6 |
| 23 | 0 | 23 | 1687,4 | 2082,9 | 4,0 | 0,9 | 26,0 | 1,7 | 0 | 73,9 |
| 24 | 1 | 40 | 24,3 | 38,6 | 2,9 | 1,9 | 20,9 | 1,8 | 0 | 57,7 |
| 25 | 0 | 18 | 42,4 | 84,9 | 4,5 | 2,0 | 8,7 | 1,7 | 0 | 62,7 |
| 26 | 1 | 24 | 85,3 | 158,6 | 3,6 | 3,3 | 7,1 | 1,7 | 1 | 86,1 |
| 27 | 1 | 17 | 6,7 | 7,6 | 1,6 | 2,2 | 8,0 | 1,4 | 0 | 50,0 |
| 28 | 1 | 40 | 12,2 | 20,7 | 3,1 | 2,2 | 18,0 | 1,9 | 0 | 44,9 |
| 29 | 1 | 25 | 52,7 | 127,1 | 4,8 | 2,4 | 10,2 | 1,7 | 0 | 40,0 |
| 30 | 1 | 18 | 146,6 | 1591,6 | 18,1 | 1,8 | 10,3 | 1,7 | 1 | 7,6 |
| 31 | 0 | 27 | 156,2 | 232,1 | 2,8 | 1,9 | 14,0 | 1,8 | 0 | 0,1 |
| 32 | 0 | 24 | 79,5 | 68,6 | 1,9 | 1,9 | 12,5 | 1,7 | 0 | 57,2 |
| 33 | 1 | 19 | 0,0 | 0,0 | 0,0 | 1,8 | 10,6 | 0,0 | 0 | 31,4 |
| 34 | 0 | 43 | 148,7 | 599,5 | 7,8 | 2,3 | 18,7 | 1,8 | 1 | 6,0 |
| 35 | 0 | 25 | 222,7 | 1043,8 | 9,2 | 1,9 | 13,4 | 1,7 | 0 | 60,9 |
| 36 | 1 | 28 | 27,8 | 44,7 | 2,8 | 1,8 | 15,6 | 1,7 | 0 | 67,7 |
| 37 | 1 | 21 | 182,5 | 834,4 | 12,6 | 1,5 | 13,7 | 1,7 | 1 | 5,7 |
| 38 | 1 | 45 | 0,0 | 0,0 | 0,0 | 2,0 | 0,0 | 0,0 | 1 | 55,5 |
| 39 | 0 | 17 | 38,4 | 60,1 | 2,7 | 1,9 | 8,9 | 1,7 | 0 | 73,2 |
| 40 | 1 | 53 | 207,5 | 497,4 | 14,6 | 1,9 | 27,9 | 1,7 | 1 | 12,4 |
| 41 | 1 | 55 | 56,8 | 111,2 | 3,9 | 2,0 | 27,5 | 1,8 | 0 | 53,6 |
| 42 | 1 | 38 | 174,5 | 327,7 | 9,1 | 2,4 | 16,2 | 1,6 | 0 | 31,4 |
| 43 | 1 | 24 | 0,0 | 0,0 | 0,0 | 2,0 | 12,0 | 0,0 | 0 | 102,5 |
| 44 | 0 | 15 | 7,2 | 4,2 | 1,1 | 1,2 | 12,2 | 1,6 | 0 | 59,2 |
| 45 | 0 | 34 | 202,2 | 548,8 | 6,4 | 2,0 | 17,0 | 1,7 | 1 | 14,7 |
| 46 | **0** | 26 | 48,9 | 49,4 | 1,7 | 1,6 | 16,0 | 1,7 | 0 | 74,4 |
| 47 | 0 | 46 | 94,7 | 520,6 | 15,6 | 2,5 | 18,4 | 1,7 | 1 | 7,2 |
| 48 | 0 | 44 | 444,0 | 990,1 | 3,9 | 2,2 | 20,0 | 1,8 | 0 | 29,7 |
| 49 | 0 | 43 | 0,0 | 0,0 | 0,0 | 1,1 | 39,1 | 0,0 | 0 | 5,2 |

**Table S1**. All relevant data.
